# Giant Virus-Eukaryote Interactions as Ecological and Evolutionary Driving Forces

**DOI:** 10.1128/mSystems.00737-21

**Published:** 2021-08-24

**Authors:** Chuan Ku

**Affiliations:** a Institute of Plant and Microbial Biology, Academia Sinica, Taipei, Taiwan

**Keywords:** amoeba, omics, gene content, microalga, *Nucleocytoviricota*, protist, virus-host interactions

## Abstract

Giant DNA viruses of eukaryotes are notable for their extraordinary genome size and coding capacity. Once thought to be oddities in the virus world, these elusive microbes have turned out to be widely occurring in marine, freshwater, and terrestrial ecosystems and are commonly associated with diverse hosts, in particular microbial eukaryotes. This commentary discusses how new sequencing techniques and information can inform us about the interactions between giant viruses and eukaryotic hosts during the viral replication cycle and their implications for ecological and evolutionary processes across different spatiotemporal scales.

## COMMENTARY

Viruses are traditionally defined as small infectious biological entities. However, a rapidly increasing number of viruses with large particles and genomes are associated with diverse eukaryotes, including vertebrates, invertebrates, algae, and various other protists from across the eukaryotic tree of life (1). These nucleocytoplasmic large double-stranded DNA viruses (NCLDVs) are classified as the phylum *Nucleocytoviricota* (https://talk.ictvonline.org/taxonomy). While NCLDVs vary along a size continuum, their relatively gigantic dimensions justify the use of giant viruses (GVs) to refer to this group of viruses with shared characteristics and genes.

Except for smallpox, African swine fever, and some deadly diseases in fish, tetrapods, and insects, most of the seven or so GV families recognized to date—major clades in phylogenetic trees—infect only or predominantly microorganisms. Microbial, mostly unicellular, eukaryotes compose the vast majority of eukaryotic taxa and exhibit immense diversity in organelles, cellular structures, and life cycles. Although they make up less than 1% of the biomass on our planet (2), they can pose public health concerns and exert primary roles in ecosystem functioning across a wide range of aquatic, terrestrial, artificial, and host-associated environments. GV infections of microbial eukaryotes can thus have important implications for our environment.

## CHALLENGES AND OPPORTUNITIES IN HIGH-THROUGHPUT METHODS

**FIG 1:**
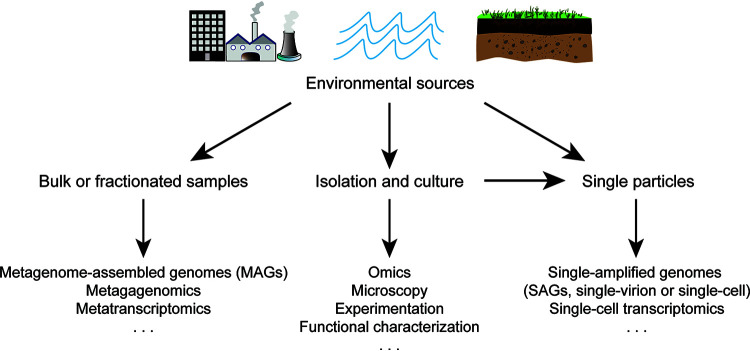
Approaches to studying giant viruses in the environment. Interactions between giant viruses and eukaryotic hosts in diverse ecosystems can be examined using culture-dependent methods or culture-independent meta-omic and single-particle sequencing.

Approaches to studying GVs encompass culture-dependent and culture-independent methods (Fig. 1). Since the 1970s, GVs have been observed from a number of microbial eukaryotes in the field, isolated, and cultured in the lab. Because of their experimental tractability, these cultured viruses are among the better studied GVs, such as chloroviruses infecting green algae that are often endosymbionts in ciliates and other eukaryotes (3). Most of the GVs, however, are only known as partial genomes from environmental sequencing data of mixed community samples. These metagenome-assembled genomes (MAGs) allow us to tap into the genomic diversity of GVs in uncultured samples (4). In addition, metatranscriptomic sequencing of expressed transcripts can target the functional repertoires of uncultured GVs (5). Mounting evidence suggests that GVs are the major component of the environmental eukaryotic virome at both the genomic and transcriptomic levels.

A major disadvantage of meta-omic approaches is their bulk sampling nature that prevents tracing a particular genomic fragment back to a specific cell or organism. Cooccurrence analyses can help predict the eukaryotic taxa of GV hosts (6), but the exact hosts remain elusive due to the complex composition of communities. An alternative approach is single-particle sequencing that can be applied to individual virions (7) or cells (8). The sequencing of single cells isolated by fluorescence-activated cell sorting (FACS) avoids chimeric contigs stemming from different cells, and if a GV genome, possibly in multiple copies, is inside a cell, a direct GV-host link can be established. Single-cell RNA-sequencing (scRNA-seq) can additionally profile the transcriptomic dynamics during GV infection (9). In the Ku lab, we are developing an approach combining single-cell genomic and transcriptomic sequencing to study GV-host interactions in different environments. While DNA fragments can indicate the existence and abundance of GVs and other associated microbes, scRNA-seq profiles gene expression patterns of the interacting partners (10). In addition to host range of uncultured GVs, it could be useful to reveal complex virus-host relationships (e.g., GV infection of symbionts in other eukaryotes) and how gene expression of a GV varies with hosts, associated microbes, or environmental settings. Using this approach, we aim to provide snapshots of GV-eukaryote interactions in diverse environments and long-term observations of their dynamics in selected sites.

Despite the relative ease of acquiring omic data, many aspects of viral biology cannot be studied without lab cultures. It is therefore important to stress the need for high-throughput protocols for systematic isolation and culture of GVs, for example, with the use of amoebae that enable the replication of a wide range of GVs (11) or a customized panel of culturable eukaryotes from a potential GV source environment. Given that MAGs represent the majority of GVs we know today, reverse genomics and targeted isolation (12) can also be useful techniques to study selected GVs based on their MAG sequences.

## GIANT VIRUS-EUKARYOTE INTERACTIONS IN ECOLOGICAL AND EVOLUTIONARY CONTEXTS

**FIG 2:**
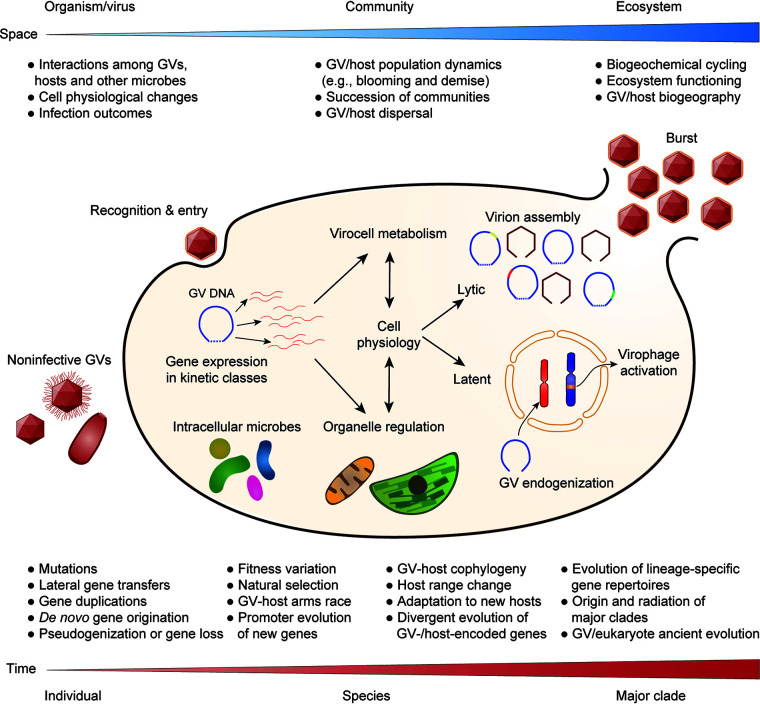
Schematic overview of known and putative interactions between giant viruses and eukaryotes, as well as the associated ecological and evolutionary processes and consequences.

The replication cycle of GVs involves virus entry, gene expression, DNA replication, virion assembly, and release (Fig. 2). Typical virion release causes cell lysis and dramatic decline of host populations, which is a crucial mechanism for demise of massive annual coccolithophore algal blooms and thus for global carbon cycling (13). However, release of virions through exocytosis has also been observed in different GV-host systems (14). In addition, it was shown that a long-term latent period of temperate infection can precede the typical lytic cell burst, which is induced by host physiological stress (15), suggesting much more delicate dynamics in GV-host interactions.

GV entry into host cells remains poorly understood. For chloroviruses, host recognition seems to depend on cell wall polysaccharide composition and causes differential attachment to host and nonhost cells (3). Host entry can be a major determinant of GV host range and might explain why certain phagocytic eukaryotes, including *Acanthamoeba* and *Vermamoeba*, are permissive to diverse GVs, although they are not necessarily the natural hosts of these GVs (11). In addition, the infection outcome can be determined by host antiviral pathways (16) or virophage that integrates into the eukaryotic genome as an antiviral system and is reactivated upon infection by its specific GV (17). Characterization of GV host range through the aforementioned approaches is the prerequisite for understanding host switching, virus-host arms race, and other aspects of GV biology.

Remodeling of cellular transcriptomes is another feature during GV infection. By avoiding the drawback of bulk sequencing in averaging out intercellular differences, scRNA-seq helped reveal differential shutdown of nucleus-, chloroplast-, and mitochondrion-encoded transcripts in coccolithophore cells infected by coccolithovirus (9). The cellular transcriptome is taken over by virus-encoded transcripts that come in successive waves of genes (kinetic classes) tightly regulated by promoter elements, with RNA and DNA polymerase transcripts followed by capsid protein and packaging ATPase (9).

DNA replication and virion assembly are steps where variation among virions can be generated. Although most GV genes seem to be under purifying selection, experimental evolution studies have found that gene loss and duplications can happen rapidly when factors such as culture microbiome (18) or anti-defense genes (19) are changed. Putative lateral gene transfers from hosts or associated intracellular microbes have also been reported for various GV lineages (1). These processes and *de novo* gene creations together shape the dynamic gene repertoires of GVs, but it is unknown how promoters of new genes arise and help them integrate into the suitable kinetic class. We expect the high resolution of scRNA-seq will help better resolve the kinetic classes in different GV-host systems and contribute to our understanding of promoter and gene expression evolution across GVs.

GVs can also go into latency instead of virion assembly and lysis, and there is a chance that their genomes can integrate into host chromosomes and be passed down in the host lineage, as suggested from widespread GV endogenizations in green algae (20). The biological role of GV endogenization is unclear, and it remains to be tested whether such processes have cumulative effects on the eukaryotic gene repertoires as do genes that arose through organellar endosymbioses (21). It is possible that integrated GV genomic fragments are similar to abundant nuclear copies of modern plastid and mitochondrial DNA (NUPTs and NUMTs) that quickly become pseudogenized (22).

## GIANT VIRUSES AS EVOLUTIONARY SANDBOXES FOR CELLULAR GENES

One of the biggest surprises from GVs is their coding of genes normally found in only cellular organisms (1). In addition to translation machinery, genes apparently unrelated to viral replication—auxiliary metabolic genes—can potentially reprogram infected cells into a physiological-metabolic state called virocell (23). These genes can encode proteins absent in the host or homologs of host-encoded proteins. In the latter case, the GV homologs often have different biochemical properties that could be advantageous for viral replication. In addition, GV genes (e.g., viral rhodopsins [8]) tend to compose a separate phylogenetic clade divergent from cellular homologs. These observations suggest that the GV homologs went on their separate evolutionary path and explored a different part of the protein landscape that could eventually improve virus fitness.

One research focus in our lab is to elucidate GV gene content variation and evolution by taking into account the effects of different processes (Fig. 2) and host associations through comparative analyses. A better understanding of the gene repertoire would shed light not only on host range evolution, but also on origins of major GV clades and their implications for the debate on GV origin(s) (24). One such clade of particular interest is the family *Mimiviridae* (*Imitervirales*), likely representing the largest evolutionary radiation of GVs known so far. It is highly abundant in aquatic environments (4, 6), has greater taxon richness than Bacteria and Archaea (25) and the highest host diversity among GV families (1, 6), and yet has lower within-family sequence divergence than several other GV families in gene trees (4, 8).

In summary, recent advances in understanding GV-eukaryote relationships are fundamentally changing our view of the associated ecological and evolutionary processes at all levels (Fig. 2). Although we are only beginning to grasp their diversity, it is exciting to witness how our knowledge of GVs has been growing. By combining newly developed methods in systems microbiology and other fields (Fig. 1), there is great potential for better comprehending their functional roles and answering challenging questions about their ecology and evolution, which are also relevant to our global environment and biological resources.
